# Protocol for the implementation and evaluation of a community-based intervention seeking to reduce dietary salt intake in Lithgow, Australia

**DOI:** 10.1186/1471-2458-14-357

**Published:** 2014-04-14

**Authors:** Mary-Anne Land, Paul Jeffery, Jacqui Webster, Michelle Crino, John Chalmers, Mark Woodward, Caryl Nowson, Wayne Smith, Victoria Flood, Bruce Neal

**Affiliations:** 1 The George Institute for Global Health, Sydney Medical School, The University of Sydney, Missenden Road, Po Box M201, Camperdown, NSW 2050, Australia; 2 Deakin University, Melbourne, Australia; 3 Faculty of Health Sciences, New South Wales Health, Sydney, Australia; 4 The Sydney University of Sydney and St Vincent’s Hospital, Sydney, Australia; 5 Royal Prince Alfred Hospital, Sydney, Australia

**Keywords:** Salt, Sodium, Community-based intervention, Cardiovascular disease prevention

## Abstract

**Background:**

Excess dietary salt is a leading risk for health. Multiple health, government, industry and community organisations have identified the need to reduce consumption of dietary salt. This project seeks to implement and evaluate a community-based salt reduction intervention.

**Methods:**

The study comprises a baseline assessment followed by a targeted intervention and then an evaluation of efficacy. The study location is Lithgow, a regional town in New South Wales, Australia. The salt reduction intervention is based upon the Communication for Behavioural Impact framework which utilises an integrated communication model to enact community advocacy and impact by providing tools that enable the translation of knowledge into behavioural change. The duration of the intervention will be between 6 and 12 months. The primary evaluation will be through measurement of 24-hr urinary sodium excretion in independent population samples aged > 20 years, drawn before and after the intervention period. The study is designed to detect a difference in mean sodium excretion of 0.7 grams per day or greater with 80% power and p = 0.05.

**Discussion:**

This study will provide a robust evaluation of the effectiveness of a community-based intervention seeking to reduce dietary salt intake using the Communication for Behavioural Impact framework. The results will provide important new evidence to inform the design and implementation of current and future salt reduction policies in Australia. The results will also have important international implications because, following the recent World Health Organization recommendations for the control of non-communicable diseases, many countries are now seeking to achieve a reduction in average population salt consumption.

**Trial registration:**

ClinicalTrials.gov, NCT02105727

## Background

The global burden and threat of non-communicable disease (NCDs) creates a major public health challenge that undermines social and economic development throughout the world [1]*.* In 2008 an estimated 63 per cent (36 million) of all global deaths were due to NCDs, comprising mostly cardiovascular diseases [2]. In Australia, cardiovascular disease is the leading cause of death and disability and the number one cost to the health sector [3,4]. There is clear evidence that a diet high in salt is associated with increased blood pressure, and that increased blood pressure leads to increased risk of vascular diseases [5]. Whilst there is no definitive estimate of population dietary salt intake in Australia, it is widely accepted that average consumption is between 7 and 12 g/day [6]; this is far above the suggested dietary target for Australians of 4 g/day [7].

The reduction of salt intake and sodium content of food has been recommended as a cost effective action that should be undertaken immediately, with expected accelerated results in terms of lives saved, cases of disease prevented and costs avoided [8]. As such, salt reduction activities are being promoted worldwide as a core component of efforts to achieve a 25 per cent reduction in avoidable NCDs by the year 2025. Specifically, the goal is to achieve a 30 per cent reduction in average salt consumption by 2025 [9]. The current Australian approach to reducing population salt intake is based upon voluntary food supply reformulation [10]. Community-based approaches may also be of value, especially those which place strong emphasis on behaviour impact by providing tools that enable the translation of knowledge into behavioural change [11].

In this study, two tools which may assist consumers to reduce salt consumption were identified. The first is a sodium-reduced salt substitute. Not only because a salt substitute might have an important effect on salt intake for individuals using alarge amount of discretionary salt, but also because it offers a talking point for consumers to engage around. The second tool, *“FoodSwitch”*, addresses the primary contributor to salt in the diet; processed foods. This electronic tool empowers consumers to choose healthier, lower salt versions, of food types they enjoy by providing specific advice about which products to pick from the supermarket shelves.

## Methods

The project involves a baseline assessment, followed by a targeted intervention and an evaluation of efficacy. The study is being conducted in Lithgow, a regional town with a residential population of about 20,160 [12] located 140 kilometres west of Sydney, New South Wales, Australia. Work commenced in 2010 and the evaluation will conclude with surveys done in 2014.

### Study aims

The primary objective of this study is to determine the effect of a multi-faceted community-based salt reduction intervention on mean salt intake, as estimated from measures of 24-hr urinary sodium excretion made before and after the intervention is implemented. The null hypothesis to be tested is that there will be no change in sodium excretion.

The secondary objectives are to determine the effects of the salt reduction intervention, by comparing measures before and after the intervention, on (1) population knowledge, attitudes and behaviour toward salt intake and (2) the sources of sodium in the diet, as estimated from 24-hr dietary recalls.

Exploratory objectives will be to examine (1) social inclusion as a determinant of response to the intervention; (2) the potential for a volunteer sample to provide estimates of population salt consumption and changes in population salt consumption; (3) whether spot urine samples can provide estimates of population salt consumption and changes in population salt consumption and (4) the association between urinary iodine excretion and urinary sodium excretion in females aged 20–45 years.

### Participant selection and recruitment

**Figure 1:**
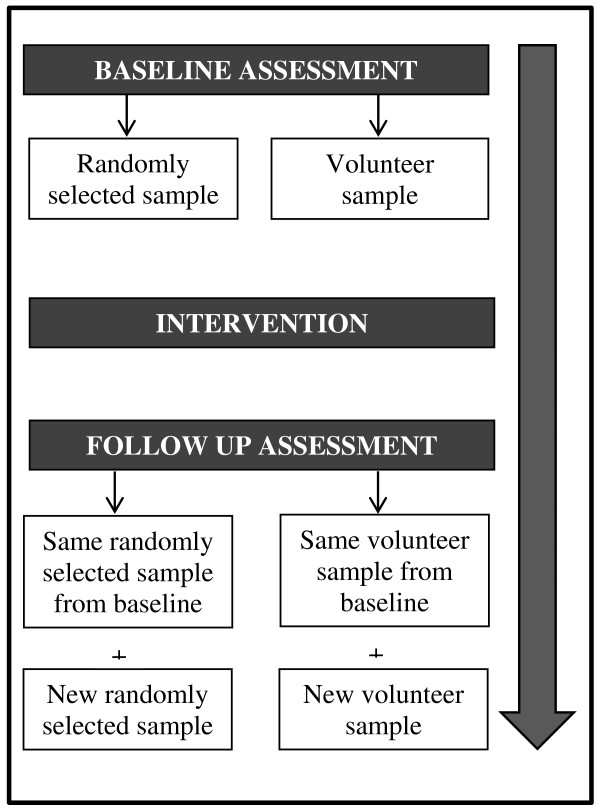
Project design.

In this study we intend to recruit two samples of individuals at baseline, a randomly selected sample and a volunteer sample. At follow up we will invite those same participants that participated at baseline, as well as recruiting an additional randomly selected sample and a new volunteer sample (Figure 1).

*For the random sample*, at baseline the electoral roll will be used to obtain the name and address of each potential participant with electronic databases searched to identify corresponding telephone numbers. At follow up a random sample will be selected from the local telephone directory. Potential participants will be first mailed invitations to take part in the survey, with an explanation of the purpose of the study, a participant information sheet and a consent form provided. These individuals will then be contacted by telephone to determine their willingness to participate and to schedule an interview time. Where a telephone number cannot be obtained, the home address will be visited by a member of the research team to determine willingness to participate.

*The volunteer sample* will be recruited by offering participation in the study to individuals at two local shopping centres, workplaces and community events. An information booth will be set up where those interested can seek further information about participation and arrange a visit by a member of the study team.

*Re-sampling of the participants included at baseline* will be done by verifying participants are still residents of Lithgow using electronic databases and then mailing invitations to take part in the follow up survey in the same way that we did for initial sampling.

### Participant inclusion and exclusion criteria

All adults over the age of 20 years residing in the Lithgow area are eligible, with no exclusion based on inter-current illness, use of medications or any other aspect of demography or personal history.

### Data collection

The data collection will be identical before and after the intervention and will be exactly the same for every participant. The only exception to this is that eight questions not included in the baseline assessment will be added to the follow up survey to measure social inclusion and to assess the proportion of the population that have been reached by the main components of the intervention program. On each occasion, the assessment will commence with a visit to the study participant by a trained research assistant. Once consent has been obtained, the five components of data collection will be initiated, comprising a questionnaire about participant characteristics, a brief physical examination, a questionnaire evaluating consumer knowledge attitudes and behaviours, a multiple pass 24-hr dietary recall and a spot and 24 hr urine collection. Both questionnaires, the physical examination and the dietary recall are completed at the time of the visit. The urine collection is scheduled for a time within the following 3–10 days and a second dietary recall, administered by telephone, will be scheduled for a random sample.

The *questionnaire* is fully structured and will be administered by a research assistant with all responses based on self-report. The questionnaire records information on socio-demographic variables, vascular disease history and current drug treatments. Participants are asked to provide the names of regular medications; if these are not known the purpose of each medication will be recorded (for example, “blood pressure lowering medication”).

The measurement of *knowledge attitude and behaviours* towards salt is based upon a questionnaire adapted from the World Health Organization/Pan American Health Organization protocol for population level sodium determination [13]. The questionnaire contains nine questions; four related to knowledge of personal consumption, recommended daily intake and possible harmful effects of salt and five assessing attitudes and behaviours to lowering salt intake. The participants answer on a range of different scales such as “rarely, sometimes, often”, “yes, no” and “too much, just the right amount or too little”.

*The physical examination* comprises measurement of body weight (using calibrated Tanita HD-357 portable electronic scales (USA)) and height (using a calibrated portable stadiometer Wedderburn WS-HRP model (Australia)) to the nearest 0.1 kg and 0.1 cm respectively, with body mass index (kg/height(m^2^)) then calculated. Blood pressure is measured using a manual inflation blood pressure monitor (A&D UA-&704) in triplicate, according to the American Heart Association protocol [14].

*The multiple pass 24-hr dietary recall* is used to determine all food and beverages consumed from midnight to midnight on the day before the interview, this method has been described in detail in prior reports [15]. Food model booklets are used to assist with the reporting of quantity and prompts provided by interviewers are used to probe for complete food descriptions, variable recipe ingredients, and food preparation including salt added during cooking and at the table. Dietary data are entered into the nutrient analysis package Foodworks Professional version 7, and analysed using AUSNUT 2007 of Food Standards Australia New Zealand. The food coding guidelines [16] are used to code each food and beverage into major, sub-major and minor food categories.

*A single 24-hr urine collection* will be obtained with the first voided urine upon waking on the day of collection being discarded and participants then collecting all voided urine up to and including the first void the following morning. The times at the beginning and the end of urine collection are recorded. During the morning of the collection period a *single morning spot urine sample* will be obtained by asking participants to collect a sample into a 30 ml container. The volumes of the spot sample and the remainder of the 24 hr urine will be recorded and each will be separately assessed for sodium, potassium, creatinine and iodine. The volume and concentration data for each sample will be combined to derive the 24-hr sodium excretion estimate for each individual. The urinary sodium concentration will be measured by ion-selective electrode with the buffered kinetic Jaffe reaction without deproteinisation used for assay of urine creatinine (Cobas Integra 400). The urinary iodine measurement will be completed by using ammonium persulfate digestion prior to Sandell-Koltoff reaction in a microtitre plate format. Suspected inaccurate urine collections (i.e. urinary creatinine < 4.0 mmol/day for women, or < 6.0 mmol/day for men, or a 24-hr urine collection of < 500 ml for either sex) and extreme outliers for urinary creatinine (> 3 standard deviations from the mean) will be excluded from the primary analysis.

### The salt reduction intervention

The goal of the intervention is to reduce salt consumption amongst adults residing in Lithgow. The means by which this will be achieved is through the application of the Communication for Behavioural Impact framework which utilises an integrated communication model to enact community advocacy and impact [17]. There are five broad components to this approach:

1. *Administrative mobilisation and public advocacy* – for putting salt reduction on the agenda of all doctors and nurses, allied health professional and local government staff via engagement through a series of meetings. The desired outcome is for these professionals to advocate for salt reduction within the community.

2. *Community mobilisation –* Businesses, workplaces and school settings will be engaged through meetings, presentations and the provision of the tools to enact salt reduction.

3. *Advertising* – Local channels of communication including newspapers, social media and radio will be targeted with stories about the program. A series of specific advertising initiatives involving local and social media will be scheduled over the period of the intervention.

4. *Interpersonal communication –* information booths will be established in the two main shopping areas and by door knocking individual homes. The tools will be supplied to support interpersonal communication and engagement across the community. These tools will support one-on-one interactions with the community and enable conversations about the importance of reducing salt consumption as well as providing practical ways to achieve a reduction. (see the ‘key messages for intervention’ subsection).

5. *Point of Service/Sale –* the salt substitute has been made available at local cafes and restaurants for use by consumers. Local bakeries have also been provided with the salt substitute to use in baking. Government buildings, medical centres and pharmacies are also stocked with the salt substitute to enable consumers to obtain and replace the salt substitute at no cost.

### Key messages for the intervention

*The salt substitute* comprises a sea salt blend of 136 mg sodium and 176 mg potassium per serving size (0.8 g). This formula results in 70% less sodium than regular salt while retaining good sensory properties [18].

*FoodSwitch* is a smartphone application which allows users to scan the barcodes of packaged foods, receive colour-coded ratings for four key food components (total fat, saturated fat, sugar and salt) and a list of similar foods that are lower salt healthier choices [19].

### Sample size

The planned sample of 600 individuals with 24-hr urine samples in the baseline and follow-up surveys will provide more than 80% power to detect a difference of 0.7 g/day salt between the mean levels of excretion before and after the intervention. There will be more than 95% power to detect a difference of 1.0 g/day salt or greater.

### Data analysis

The characteristics of the full baseline and follow up samples will be summarised and compared, as will the characteristics of the volunteers, those selected at random and those for which paired baseline and follow-up specimens are available.

For each individual, the 24-hr sodium excretion value (mmol/day) will be calculated as the concentration of sodium in the spot urine (mmol/L), multiplied by the spot urine volume (L/day) added to the concentration of sodium in the remaining urine (mmol/L) multiplied by the volume of the remaining urine (L/day). The conversion from mmol to grams will be made by dividing by 17 and the conversion from sodium (Na) to salt (NaCl) by multiplying by 2.542.

The primary comparison between baseline and follow-up levels of 24-hr urinary sodium excretion will be made using an inverse-variance weighted fixed effect pooled estimate of the effects in the sub-sets of participants. The primary analysis will be supplemented by subsidiary analyses that separately explore the baseline and follow-up differences for 1) the volunteer sample, 2) the random sample and 3) the sample for which both baseline and follow-up specimens are available. Differences will be tested using t-tests and paired t-tests. The analysis will be unadjusted unless there is evidence that the baseline and follow-up samples differ in regard to age, sex or body mass index, in which case these covariates will be included in the models.

Bland-Altman plots will be used to explore the agreement between spot urine and 24-hr urine collections, and to determine whether the agreement between the two methods of collection varies across different levels of sodium excretion.

Daily iodine intake will be estimated by multiplying the concentration of iodine in the urine measured in μg/l by 0.0235 and by body weight measured in kilograms [20]. Urinary iodine status data will be summarised as median urinary iodine excretion values and the proportions with iodine deficiency and iodine sufficiency according to the World Health Organization and the International Committee on the Control of Iodine Deficiency Disorders (ICCIDD) [21]. Mann–Whitney U tests will be used to compare differences in urinary iodine excretion according to World Health Organization urinary salt excretion target of ≥ 5 g/day and < 5 g/day, while general linear models will be used to investigate the continuous association between iodine and sodium excretion.

The level of agreement between dietary sodium assessments and 24-hr urinary sodium assessments will be investigated using Bland-Altman plots. While the associations of knowledge, attitudes and behaviours with 24-hr urinary sodium excretion will be investigated using general linear models.

Throughout, a p-value of 0.05 or less will be taken to indicate a finding unlikely to have arisen solely by chance. No corrections for multiple testing will be implemented. If appropriate, sensitivity analyses will use multiple imputation to cope with missing values.

### Ethics and dissemination

Permission to undertake the study was obtained from the Lithgow City Council and the project was approved by the University of Sydney Human Research Ethics Committee. Written consent was obtained from all participantswho were free to discontinue their participation at any time, with no explanation required. The findings of this research will be disseminated through conference presentations, peer-reviewed publications and the general media.

### Project status

The baseline community assessment was undertaken in the first and second quarters of 2011 and the follow-up assessment will be done in the first and second quarters of 2014. The intervention will be implemented from the third quarter of 2013 to the second quarter of 2014. The findings of the baseline assessment were used to inform the design of the community-based salt reduction intervention.

## Discussion

This study will provide a robust evaluation of the effectiveness of a community-based intervention seeking to reduce dietary salt intake using the Communication for Behavioural Impact framework. The results will provide important new evidence to inform the design and implementation of current and future salt reduction policies in Australia. The results will also have important international implications because following the recent WHO recommendations for the control of non-communicable diseases [9], many countries are now seeking to achieve a reduction in average population salt consumption [22,23].

In addition to making an estimate of the effectiveness of the salt reduction intervention itself, the study also seeks to address important practical issues in the research field. In particular, to quantitatively address the question of whether the accepted best method for assessing population salt intake, the collection of 24-hr urine specimens from a random sample, might be substituted with other more practical approaches [24,25]. Response rates in surveys requiring 24-hr urine collection are generally low because of the significant participant burden involved with sample collection [26-28]. This study will determine the impact of substituting 24-hr urine samples with spot urine samples and of using a volunteer rather than a randomly selected population sample. While both approaches are somewhat non-intuitive, there are now some data suggesting that in selected circumstances these methods may provide valid estimates of average population salt consumption levels and changes in salt consumption over time [29,30]. Identifying simpler, lower cost approaches to developing and monitoring salt reduction programs has been identified as a priority by the World Health Organization in its efforts to achieve more widespread uptake of national salt reduction programs [31]. The capacity to use a spot urine collection to estimate 24-hr sodium excretion has been a particular focus of recent research efforts and appears to show considerable promise [32-35] although there is need for further validation of the procedures and equations used [36].

In parallel to the global roll out of salt reduction efforts there is a need to ensure that actions to control the impact of iodine deficiency are not adversely impacted [37]. Iodisation of salt is the preferred method for iodine deficiency disorder elimination [38] and there is a theoretical risk that a highly effective salt reduction program could reduce the effectiveness of this strategy. Accordingly, it is proposed that teams working on salt reduction engage closely with those involved with the elimination of iodine deficiency disorder to coordinate efforts. Specifically, a World Health Organization consultation held last year [39], highlighted the inadequacy of current estimates of iodine consumption for many countries, including Australia, and identified the potential for joint analysis of urine samples from population surveys to address this issue. The meeting also emphasised that the objectives of both iodine deficiency elimination programs and salt reduction programs can be achieved through the collaborative implementation of policies and careful monitoring of salt and iodine levels. Specifically, it was recommended that if salt reduction efforts are so effective as to decrease iodine consumption from salt, then the levels of iodisation in salt should be increased [39]. This study will be one of the first opportunities since that meeting to implement the proposed joint approach and explore whether urinary iodine excretion varies according to World Health Organization urinary salt excretion targets and the estimated proportion of sodium derived from fortified sources of salt.

The primary evaluation done within this study benefits from before and after assessments of salt consumption based upon the preferred method of 24-hr urine collection, with the use of standard checks for completeness of the specimens based upon volume and urine creatinine excretion. The sample size is only moderate but the study, nonetheless, has the power required to detect plausible changes in salt consumption. The location of the study in a single town in a regional area of New South Wales will compromise the direct generalizability of the study findings to Australia as a whole although there are likely to be elements of the findings that are viewed as applicable to a range of other settings. The inclusion of alternate methods of estimating salt consumption and the relationship between salt and iodine consumption will provide important new evidence on the feasibility and strength of these approaches and will inform national and global guidelines in this field.

## Competing interests

B.N. is the Chairman of the Australian Division of World Action on Salt and Health and J.W. is Director of the World Health Organization Collaborating Centre on Population Salt Reduction. None of the other authors of the manuscript have any competing interests.

## Authors’ contributions

All authors contributed to the conception and design of the study. ML and BN participated in drafting the article while all authors revised it critically for content and final preparation for publication. All authors read and approved the final manuscript.

## Pre-publication history

The pre-publication history for this paper can be accessed here:

http://www.biomedcentral.com/1471-2458/14/357/prepub
